# Dcc Mediates Functional Assembly of Peripheral Auditory Circuits

**DOI:** 10.1038/srep23799

**Published:** 2016-04-04

**Authors:** Young J. Kim, Sheng-zhi Wang, Stephen Tymanskyj, Le Ma, Huizhong W. Tao, Li I. Zhang

**Affiliations:** 1 Zilkha Neurogenetic Institute, University of Southern California, Los Angeles, California, USA; 2 Department of Physiology and Biophysics, University of Southern California, Los Angeles, California, USA; 3 Department of Cell and Neurobiology, University of Southern California, Los Angeles, California, USA; 4 Neuroscience Graduate Program, University of Southern California, Los Angeles, California, USA; 5 Department of Neuroscience, Thomas Jefferson University, Philadelphia, Pennsylvania, USA

## Abstract

Proper structural organization of spiral ganglion (SG) innervation is crucial for normal hearing function. However, molecular mechanisms underlying the developmental formation of this precise organization remain not well understood. Here, we report in the developing mouse cochlea that deleted in colorectal cancer (Dcc) contributes to the proper organization of spiral ganglion neurons (SGNs) within the Rosenthal’s canal and of SGN projections toward both the peripheral and central auditory targets. In Dcc mutant embryos, mispositioning of SGNs occurred along the peripheral auditory pathway with misrouted afferent fibers and reduced synaptic contacts with hair cells. The central auditory pathway simultaneously exhibited similar defective phenotypes as in the periphery with abnormal exit of SGNs from the Rosenthal’s canal towards central nuclei. Furthermore, the axons of SGNs ascending into the cochlear nucleus had disrupted bifurcation patterns. Thus, Dcc is necessary for establishing the proper spatial organization of SGNs and their fibers in both peripheral and central auditory pathways, through controlling axon targeting and cell migration. Our results suggest that Dcc plays an important role in the developmental formation of peripheral and central auditory circuits, and its mutation may contribute to sensorineural hearing loss.

Mirror movements are intentional movements of one side of the body accompanied by mirroring involuntary movements on the other side. Mild cases of mirror movements are found in normally developing young children, but its persistency throughout adulthood is rare and found only in certain neurological disorders such as Klippel-Feil syndrome (KFS)^1^. Eight independent mutations in Dcc, leading to Dcc proteins lacking most of the functional domains, have recently been identified in different families with congenital mirror movements (CMM), which is likely to be a result of defective midline crossing of axonal fibers^1^,^2^,^3^. Mirror movement behaviors caused by mutations in Dcc are not only limited to humans but have also been observed in mice and zebrafish^4^,^5^. Clinically, mirror movement is diagnosed as a part of complex symptoms in KFS, which often accompanies sensorineural hearing loss^6^,^7^,^8^,^9^,^10^,^11^. Although no direct clinical link between KFS and Dcc mutation has been established yet, we postulate that some cases of sensorineural hearing loss observed in KFS patients with MM may involve Dcc mutation. In addition, sensorineural hearing loss has been observed in some congenital syndromes exhibiting an absence of corpus callosum (agenesis of corpus callosum, ACC)^12^,^13^, which can result from Dcc mutations^14^.

Dcc is a gene encoding the receptor for Netrin-1 (Ntn1)^15^. Dcc, originally identified in humans as a tumor suppressor gene, has been well characterized in the developing nervous system of various model organisms. Its wide variety of functions include neuronal precursor cell migration^16^, axon guidance^17^, axon branching^18^, axon innervation^19^, and oligodendroglial development^20^. A significant amount of what is known about Dcc comes from detailed studies of commissural axons in the developing spinal cord^21^, which indicate that Dcc serves as a guidance cue for commissural axons to cross the midline. Expression studies have demonstrated that all of the “classical” families of axon guidance cues are expressed in the developing ear, suggesting that a complex network of these signaling mechanisms controls cochlear development and its innervation pattern^22^. Recently, several of these signaling pathways in the developing mouse cochlea have been characterized. For example, Slit/Robo signaling regulates the spatial restriction of SGNs^23^, while various Eph/ephrin signaling molecules affect SGN neurite outgrowth and HC innervation patterns^24^,^25^,^26^. The apparently prominent developmental roles of the conserved families of axon guidance molecules in the cochlea thus prompted us to examine the contribution of Dcc in mouse cochlear development.

We first observed two defects in the cochlea of Dcc mutant mice: disrupted SGN assembly in the Rosenthal’s canal, and misrouted afferent fibers of SGNs. By tracing the changes developmentally, we found that E16.5 was the earliest time point when the disruption of SG assembly could be observed, excluding any possible origin of SGN delamination defects. Similar disruption of SGN positioning and misrouting of fibers was also observed in the central auditory pathway towards the cochlear nucleus. In addition, the bifurcation pattern of auditory nerve fibers was disrupted in Dcc mutants. Our results revealed a previously unrecognized role of Dcc in regulating the proper organization of not only SGN cell bodies but also their neurites in the developing auditory system. Such disrupted spatial patterning of SGNs and their fibers could be a causal factor that gives rise to hearing impairments seen in clinical cases involving Dcc mutations.

## Result

### Gene expression analysis of classical axon guidance molecules in mouse cochlea

Previous studies suggest involvements of classical axon guidance molecules in the developing ear^22^. To understand the expression patterns of axon guidance molecules, we have carried out a RNA-sequencing analysis among defined cochlear cell populations at postnatal stages (P3 to P7): hair cells (HCs), supporting cells (SCs) in the greater epithelial ridge (GER), and spiral ganglion neurons (SGNs) in the spiral ganglion (SG)^26^. Our results demonstrated the presence of previously unstudied guidance molecules in the inner ear (e.g. Sema6A-D, Efna5, EphA1) as well as molecules with known patterns of expression in cochlear regions (Table 1). Consistent with our previous *in situ* hybridization data^23^, Slit2 transcript was highly enriched in the SC population (Table 1). Further, the expression levels of Nrp2 and Efna5 in SGN and HC populations were consistent with their previously reported expression patterns^25^,^27^.

**Figure 1:**
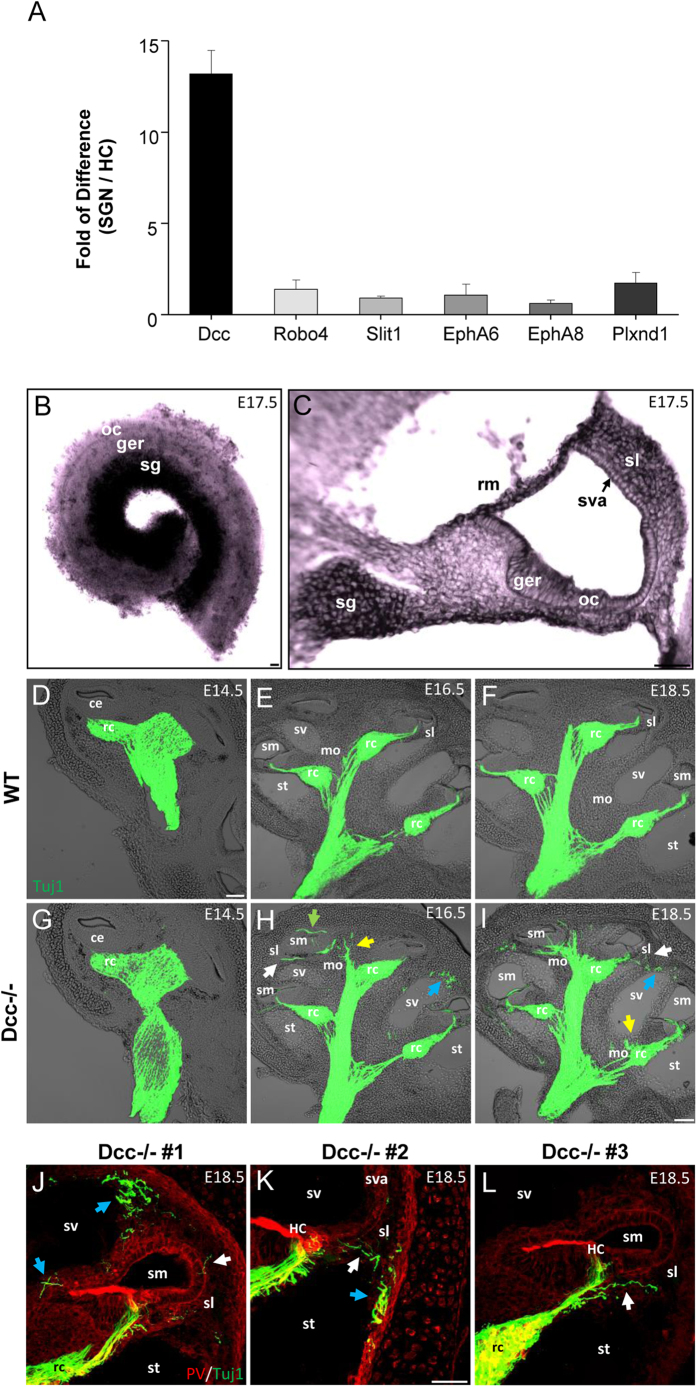
Disorganized SG fibers in the absence Dcc in SGNs. (**A**) Microarray analysis showing fold of difference between SGN and HC populations purified from P5-P7 mouse cochleae for selected axon guidance molecules based on the RNA-sequencing data. N = 3. Bar = SD. (**B**) *In situ* hybridization of Dcc in the whole-mount cochlea at E17.5. “oc”, organ of corti; “ger”, greater epithelial ridge; “sg”, spiral ganglion. (**C**) *In situ* hybridization of Dcc in the transverse section of E17.5 cochlea. “rm”, reissner’s membrane; “sl”, spiral ligament; “sva”, stria vascularis. (**D–F**) Representative images of cross-sections of wild-type cochleae at E14.5, E16.5, and E18.5, immunostained with anti-Tuj1 (green) antibody and merged with differential interference contrast (DIC) images. “ce”, cochlear epithelium; “sl”, spiral ligament; “sv”, scala vestibule; “sm”, scala media; “st”, scala timpani; “mo”, modiolus; “rc”, Rosenthal’s canal. (**G–I**) Cross-sectional images of Dcc^−/−^ cochleae, treated similarly as in (**D–F**). White arrows mark misrouted fibers in “sl”. Blue arrows mark misrouted fibers in mesothelial cells of “sv” or “st”. Yellow arrows indicate fibers in “mo”. Green arrow marks fibers in “sm”. (**J–L**) High-magnification cross-sectional views of E18.5 mouse cochlea from three Dcc^−/−^ animals, immunostained with anti-PV (red) and anti-Tuj1 (green) antibodies. Misrouted fibers are marked in similar manners as in (H,I).All scale bars represent 50 μm.

From the analysis of all families of classical axon guidance molecules (Table 1), we found that six genes were highly specifically expressed in SGNs. These genes included Dcc, Robo4, Slit1, EphA6, EphA8, and Plxnd1. From our previously generated microarray-based gene profile data also at postnatal stages^23^, we found that among these six genes, Dcc exhibited the highest fold difference between the SGN (non-sensory) and HC (sensory) populations (Fig. 1A).

### Expression of Dcc in the developing cochlea

In the auditory system, the expression pattern of Dcc has been characterized in the early embryonic and adult vestibulocochlear ganglion and in the ventral cochlear nucleus^28^,^29^,^30^. However, its expression during the peripheral auditory circuit development has not been studied. To verify the expression of Dcc in the cochlea within the crucial developmental time window when the peripheral innervations take place, we performed *in situ* hybridizations in the cochlear whole-mount and cross-sections at the embryonic stage 17.5 (E17.5). Strong Dcc signals were found in the SG, but not in the organ of Corti (oc) or greater epithelial ridge (ger) (Fig. 1B). Cross-sectional views of E17.5 cochlea revealed that Dcc was also expressed in the Reissner’s membrane (rm) and spiral ligament (sl) lateral to the stria vascularis (sva) (Fig. 1C). The overall expression pattern of Dcc transcript was consistent with our gene profiling results (Table 1). The strong expression of Dcc in the SG suggests that it may play a role in regulating the development of SGNs and their fibers.

### Misrouted SGN fibers in the absence of Dcc

Based on the known functional roles of Dcc in the developing nervous system and its highly enriched expression pattern in SGNs, we hypothesized that proper axon guidance may be provided by Dcc signaling during cochlear development. To test this hypothesis, we compared the innervation pattern of SGN fibers in cochlear cross-sections between wild-type and homozygous Dcc mutant (Dcc^−/−^) mice. The latter harbors an insertion of a neomycin resistance cassette into exon3 of Dcc gene, which prevents the production of full length proteins^14^. At E14.5, peripheral fibers of SGNs, which would innervate HCs, have not been fully extended out yet in either wild-type or Dcc^−/−^ cochleae (Fig. 1D,G). However, cross-sectional views of Dcc^−/−^ cochleae at E16.5 and E18.5 revealed that ectopic fibers were routed towards the peripheral side of the cochlea, while central auditory nerves did not show any signs of aberrant fiber trajectories (Fig. 1E,F,H,I). High-magnification views of cochlear cross-sections showed exact locations of misrouted SG fibers in ectopic places such as the modiolus (mo) near the Rosenthal’s canal (rc) (Fig. 1H,I), spiral ligament (sl), and mesothelial cells of scala vestibule (sv) and scala tympani (st) (Fig. 1J–L), whereas wild-type tissues showed no traces of Tuj1 signals in any of these regions (Fig. 1F).

**Figure 2:**
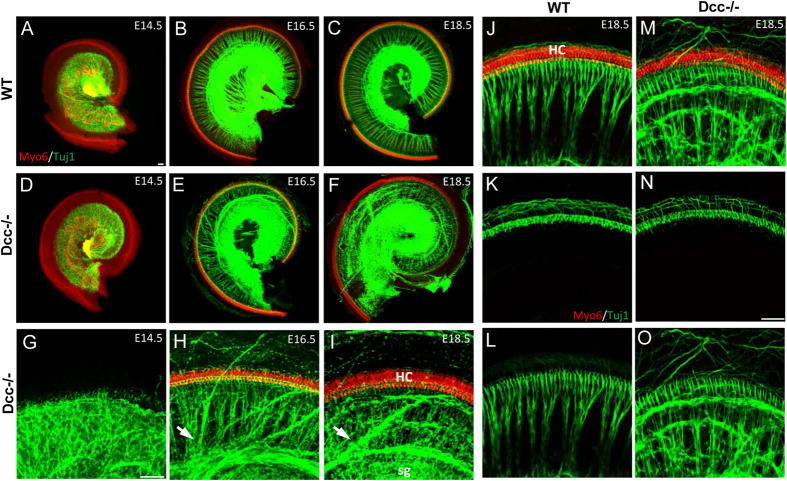
Defective peripheral innervation patterns in Dcc^−/−^ mice. (**A–C**) Representative images of whole-mount wild-type cochleae at E14.5, E16.5, and E18.5, immunostained with anti-Myo6 (red) and anti-Tuj1 (green) antibodies. (**D–F**) Images of whole-mount Dcc^−/−^ cochleae, stained in similar manners as in (**A–C**). (**G–I**) Higher magnification images of cochleae shown in (**D–F**). Arrows point to misrouted fiber bundles originating from SG. “HC”, hair cells; “sg”, spiral ganglion. (**J–O**) Representative z-stack projections images of the base region of wild-type and Dcc^−/−^ cochleae at E18.5, immunostained with anti-Myo6 (red) and anti-Tuj1 (green) antibodies. Images in (**K,N**) are the projection of a subset of z-stack images from (**J,M**), showing only the HC layers, while images in (**L,O**) exclude stacks for HC layers. All scale bars represent 50 μm.

Misrouted fibers in the peripheral side of the cochlea prompted us to closely examine the pattern of SGN innervation of HCs. At E14.5, when inner radial bundles (i.e. fasciculated afferent/efferent fibers) were not yet visible in a whole-mount preparation, there was no apparent difference in the overall morphology between Dcc^−/−^ and wild-type cochleae (Fig. 2A,D,G). At E16.5 and E18.5, misrouted fibers originating from the SG region were observed in Dcc^−/−^ but not wild-type cochleae, with some of them apparently overshooting past the sensory epithelium (Fig. 2B,C,E,F). High-magnification images showed that the misrouted fibers were usually in bundles (Fig. 2H,I). Despite the randomly misrouted fiber bundles, the overall organizational pattern of inner radial bundles was preserved to some degree throughout the whole cochlear length (Fig. 2B,C,E,F).

Next, we examined the SGN innervation pattern in the sensory epithelium. The entire z-stack images of whole-mount cochlea at E18.5 (Fig. 2J,M) were partitioned into two sets, with one consisted of planes from the apex to base of HCs (Fig. 2K,N) and the other from the base of HCs to SGN cell bodies in the Rosenthal’s canal (Fig. 2L,O). These images revealed largely normal organization of type I and type II fiber innervation of HCs in the Dcc mutant, as no aberrant fibers were observed around the HC rows (Fig. 2K,N). However, fibers innervating HCs were less densely packed in the Dcc^−/−^ mutant (Fig. 2K,N; percentage area occupied by fibers in HC regions = 4.05% ± 0.78 for WT and 2.76% ± 0.45 for Dcc^−/−^, p < 0.01, t test, N = 8 embryos for both genotypes). All the misrouted fibers in the Dcc^−/−^ mutant were observed in the z-stack image representing the space below the HC layers (Fig. 2L,O), indicating a failure of these fibers to enter their target area.

### Reduced ribbon synapses due to misrouted afferent fibers in the Dcc^−/−^ mutant

**Figure 3:**
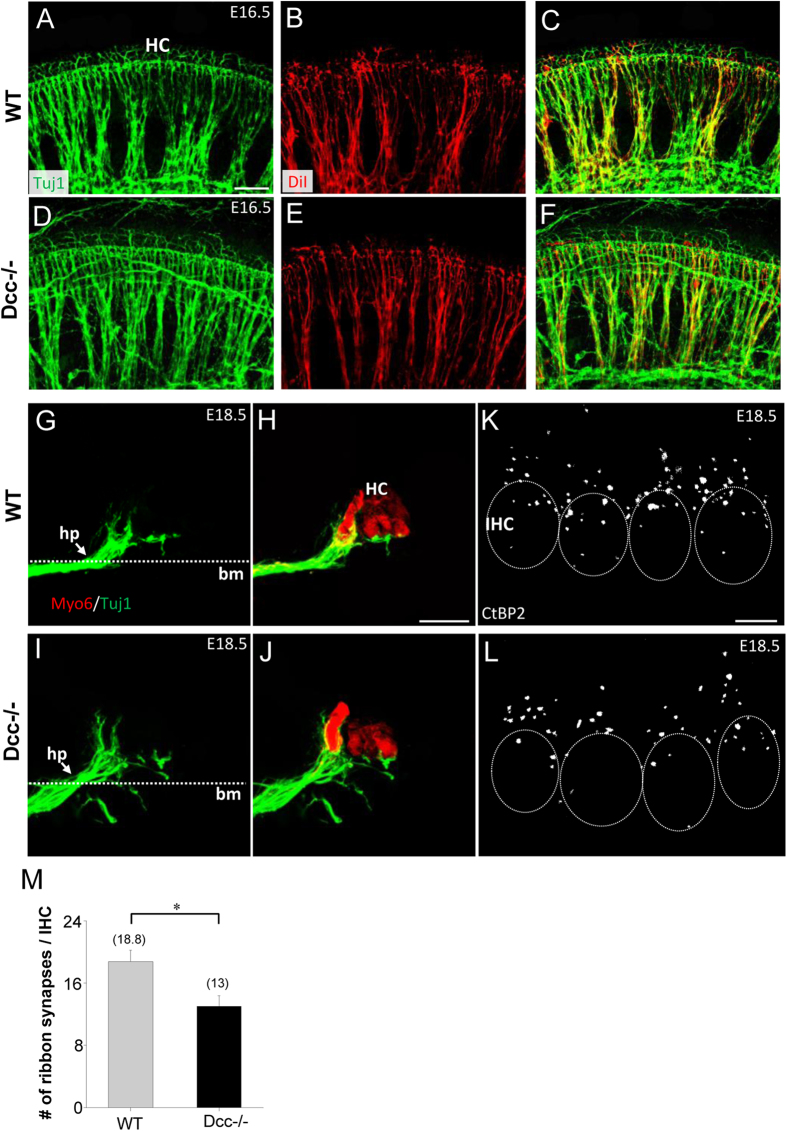
Diminished afferent synaptic connections in the Dcc mutant cochlea. (**A–F**) Representative images of whole-mount wild-type and Dcc^−/−^ cochleae at E16.5, immunostained with anti-Tuj1 (green) antibody and labelled with DiI for efferent fiber projection patterns and their superimposed images. (**G–J**) Representative images of cross-sections of the mid region of E18.5 wild-type and Dcc^−/−^ cochleae, immunostained with anti-Myo6 (red) and anti-Tuj1 (green) antibodies, showing SG fibers innervating HCs. Images of SG fibers (green) and HCs (red) are superimposed in (**H,J**). “bm”, basilar membrane; “hp”, habenula perforate. (**K,L**) Whole-mount images from the mid-base region of wild-type and Dcc^−/−^ cochleae at E18.5, immunostained with anti-CtBP2 antibody. Each white spot labels one ribbon synapse. Dotted ovals indicate individual inner hair cell (IHC) nuclei. (**M**) Average number of ribbon synapses per IHC at E18.5. Data are shown as mean ±  SEM. *p < 0.05, t test. N = 4 cochleae for both genotypes. Scale bar represents 50 μm in (**A**), 20 μm in (**H**), and 5 μm in (**K**).

Anatomical studies in the ear have shown that efferent fibers, originating from the superior olivary complex (SOC), descend via the inferior vestibular nerve and eventually travel through the Rosenthal’s canal into the organ of Corti^31^. In order to test the possibility of efferent projections contributing to the disorganization of cochlear fibers observed in the Dcc mutant, the pattern of efferent fibers was revealed by depositing a lipophilic dye, DiI, in the path of olivocochlear bundles^32^. No apparent misrouting of efferent fibers was observed in Dcc^−/−^ cochleae (Fig. 3A–F), indicating that the misrouted fibers revealed by Tuj1 staining (Fig. 3A,D) were all afferent fibers originating from SGN somas. Together, these data suggest that Dcc is important for the proper projection of afferent fibers.

We next asked whether the diminished fiber density in cochlear epithelial region (Fig. 2K,N) and ectopic projection of afferent fibers (Fig. 2L,O) in the Dcc^−/−^ mutant could result in a reduction of synapse numbers between SGNs and hair cells. We first closely examined SGN peripheral fibers that entered the organ of Corti. Past the habenula perforate (hp), a tiny opening in the spiral lamina that allowed fibers to pass to enter the organ of Corti, disorganization of fibers could still be observed in the terminal region near HCs (Fig. 3G–J). We then quantified the number of ribbon synapses in wild-type and Dcc^−/−^ cochleae, by staining with CtBP2 ref. 33 (Fig. 3K,L). Comparison of sensory epitheliums at E18.5 revealed reduced number of ribbon synapses in the Dcc^−/−^ mutant (Fig. 3M). These data further support the notion that the disruption of Dcc function impairs correct targeting of SGN fibers to form afferent synapses on HCs.

### Loss of spatial restriction of SGNs within the Rosenthal’s canal in the Dcc^−/−^ mutant

**Figure 4:**
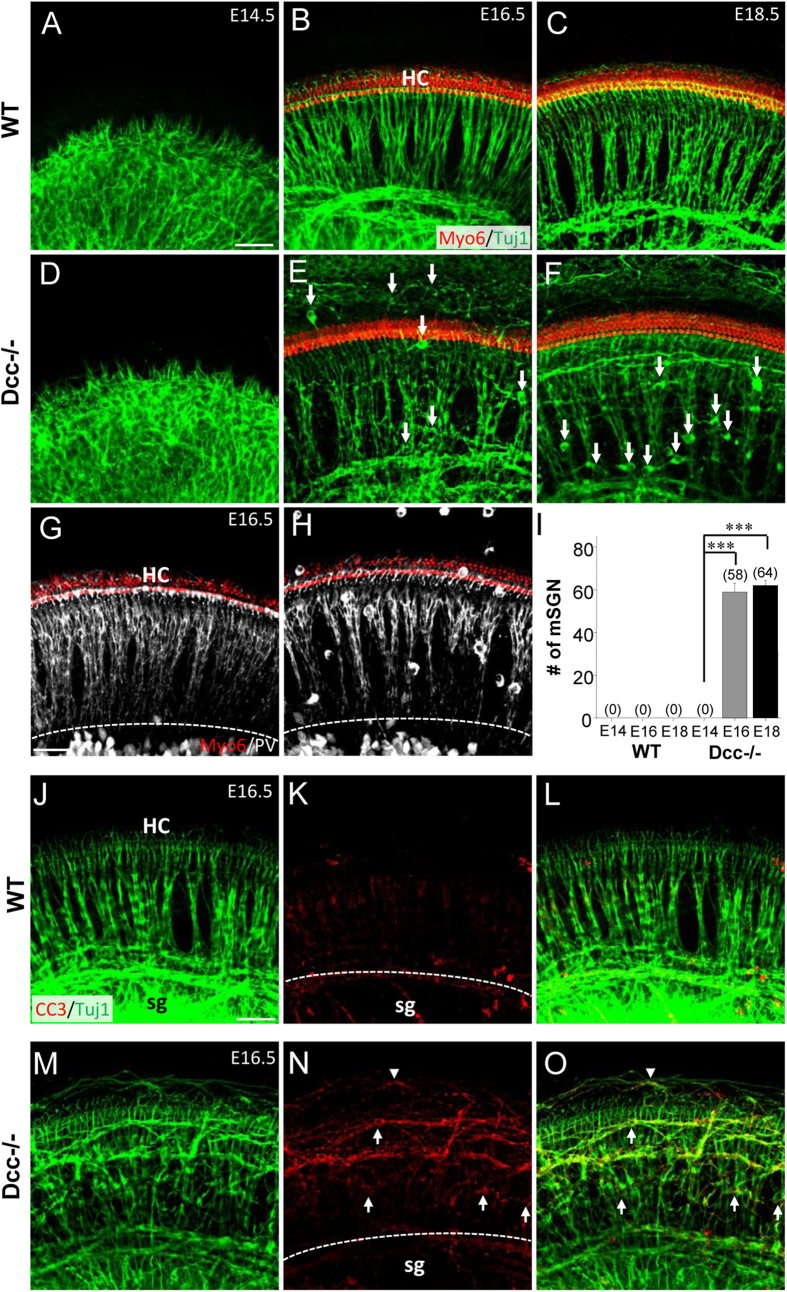
Mispositioned SGNs in the Dcc mutant cochlea. (**A–F**) Representative images of whole-mount wild-type and Dcc^−/−^ cochleae at E14.5, E16.5, and E18.5, immunostained with anti-Myo6 (red) and anti-Tuj1 (green) antibodies. White arrows point to mispositioned SGN cell bodies. (**G,H**) Whole-mount E16.5 wild-type and Dcc^−/−^ cochleae, immunostained with anti-Myo6 (red) and anti-PV (white) antibodies. White dotted curve labels the lateral boundary of SG. (**I**) Average number of mispositioned SGN cell bodies per cochlea between in wild-type and Dcc mutant animals at different developmental time points. Data are shown as mean ±  SEM. ***p < 0.001, t test. N = 8 embryos per group for both genotypes. (**J–O**) Representative images of whole-mount wild-type and Dcc^−/−^ cochleae at E16.5, immunostained with anti-CC3 (red) and anti-Tuj1 (green) antibodies. Superimposed images of SG fibers and CC3 positive signals are shown in (**L,O**). Arrows point to selected mispositioned SGN cell bodies showing CC3 labeling. Arrowhead points to selective misrouted SG fibers showing CC3 labeling. White dotted curve labels the lateral boundary of SG. All scale bars represent 50 μm.

Since Dcc has been implicated in regulating neuronal migration in various systems such as the spinal cord, forebrain, midbrain, bowel and pancreas^34^,^35^,^36^,^37^, we further examined its potential role in regulating SGN migration. In E14.5 Dcc^−/−^ cochlea, SGNs remained within the Rosenthal’s canal without any noticeable abnormalities as compared with wild-type cochleae (Fig. 4A,D). However, starting from E16.5 and continuing into E18.5, a number of ectopically located SGNs were distributed throughout the cochlea along both medial-lateral and basal-apical axis, although the majority of SGNs were still correctly located within the Rosenthal’s canal (Fig. 4B,C,E,F). We quantified the number of mispositioned SGN cell bodies by staining SGNs with parvalbumin (PV)^23^ (Fig. 4G,H). E16.5 and E18.5 mutant cochleae exhibited a comparable number of mispositioned SGNs (Fig. 4I). To determine whether the mispositioned SGNs were possibly dying neurons being separated from the intact SG, we compared the density of apoptotic neurons between wild-type and Dcc^−/−^ cochleae, using anti-Cleaved Caspase3 antibody^38^. There were no enhanced apoptotic signals within or around the SG region (Fig. 4J–O). However, signs of enhanced apoptosis were visible in the already misplaced SGN cell bodies and fibers in the Dcc^−/−^ mutant (Fig. 4K,N). These data suggest that the partial disassembly of SG in the absence of functional Dcc is not caused by cell death.

**Figure 5:**
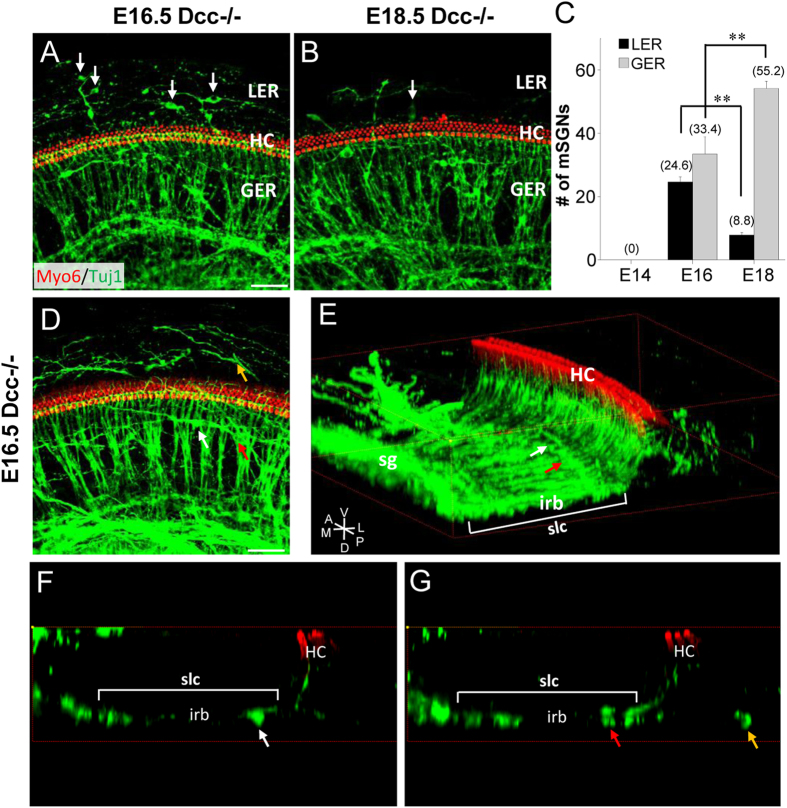
Developmental changes of SGN positions in the Dcc mutant cochlea. (**A,B**) Representative images of whole-mount Dcc^−/−^ cochlea at E16.5 and E18.5, immunostained with anti-Myo6 (red) and anti-Tuj1 (green) antibodies. Arrows mark SGN cell bodies located in the lesser epithelial ridge (LER). (**C**) Average number of mispositioned SGN cell bodies in LER and GER regions per cochlea in Dcc mutants at different time points. Data are shown as mean ±  SEM. **p < 0.01, t test. N = 8 embryos per group. (**D**) Representative z-stack projection image of whole-mount wild-type cochleae at E16.5, immunostained with anti-Myo6 (red) and anti-Tuj1 (green) antibodies. (**E**) 3D reconstructed 30° angled transverse z-stacked image of (**D**). “HC”, hair cells; “sg”, spiral ganglion; “irb”, inner radial bundle; “slc”, spiral laminal canal; V, ventral; D, dorsal; A, anterior; P, posterior; M, medial; L, lateral. (**F,G**) Transverse views from the 3D image in (**E**) at horizontal planes of the indicated mispositioned SGNs by arrows in (**D,E**). Arrows of the same color indicate same mispositioned SGN cell bodies shown in (**D–G**). All scale bars represent 50 μm.

The spatial distribution pattern of mis-migrated SGNs appeared to change developmentally. For example, there were significantly fewer mispositioned SGN cell bodies in the lesser epithelial ridge (LER), but more mispositioned SGNs in the greater epithelial ridge (GER), at E18.5 than E16.5 (Fig. 5A–C). For a systematic analysis of the location of ectopic SGNs, we reconstructed three dimensional (3D) images from confocal z-stacks. The 3D reconstructed images revealed that every single mispositioned SGN cell body ended up in the space dorsal to HC layers regardless of their lateral-medial positions (Fig. 5D–G). The mispositioned SGNs medial to HC rows were located within the spiral lamina canal (slc) where inner radial bundles (irb) passed through (Fig. 5F,G, white and red arrows). The SGN cell bodies in the LER (which is lateral to HC rows) were located on the same dorsal-ventral plane as the inner radial bundles (Fig. 5G, yellow arrow). Therefore, none of the mispositioned SGNs occupied the space within the organ of Corti per se.

### Inappropriate SGN exit from the Rosenthal’s canal towards the CNS

**Figure 6:**
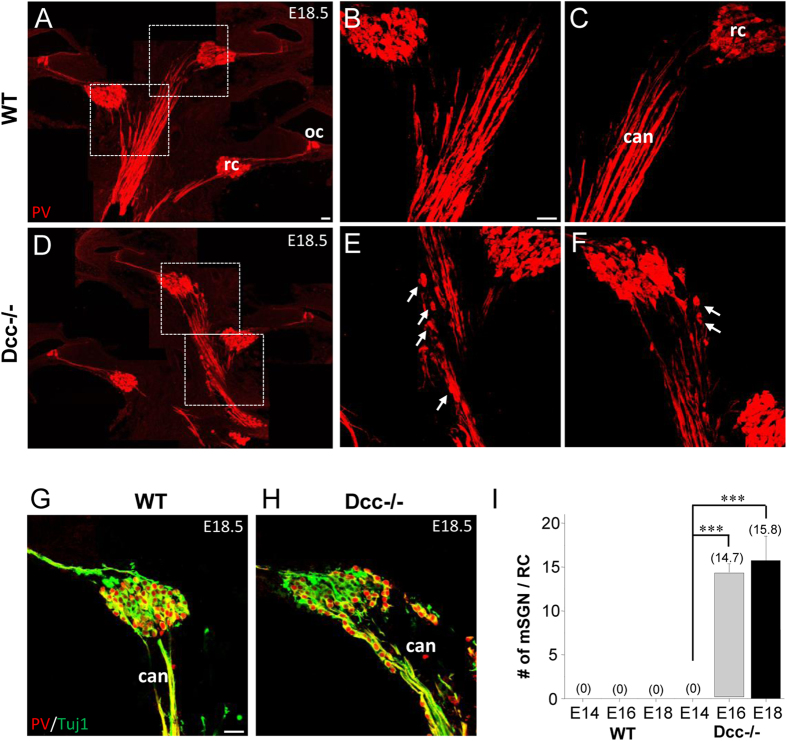
Atypical exit of SGNs from the Rosenthal’s canal along the central auditory pathway in the Dcc mutant. (**A–F**) Cross-sectional images of E18.5 wild-type and Dcc^−/−^ cochleae, immunostained with anti-PV (red) antibody. Images in middle and right panels are high-magnification views of the regions marked with white dotted squares. Arrows point to some of mispositioned SGN cell bodies. “oc”, organ of corti; “rc”, Rosenthal’s canal; “can”, central auditory nerve. (**G,H**) Cross-sectional views of the Rosenthal’s canal region of a wild-type and Dcc^−/−^ cochlea at E18.5, immunostained with anti-PV (red) and anti-Tuj1 (green) antibodies. (**I**) Average number of mispositioned SGN cell bodies within 200 μm distance from the Rosenthal’s canal along the central auditory nerve. Data are shown as mean ±  SEM. ***p < 0.001, t test. N = 6 embryos per group for both genotypes. All scale bars represent 30 μm.

As a previous report has demonstrated the importance of Dcc in confining spinal interneuron cell bodies and their axons within the central nervous system (CNS)^39^, we hypothesized that the proper organization of the central auditory pathway may also require Dcc. To test this hypothesis, we examined the SG assembly in the Rosenthal’s canal in cross-sections of Dcc mutant cochleae. In E18.5 wild-type cochleae, there was no single SGN cell body observed outside the Rosenthal’s canal (Fig. 6A–C,G). However, in the Dcc mutant, some SGN cell bodies drifted away from the Rosenthal’s canal along the pathway of the central auditory nerve (can) (Fig. 6D–F,H). The onset of ectopic SGNs in the central auditory nerve pathway (E16.5) was comparable to that in the periphery (Fig. 6I). At E14.5, SGNs were properly confined within the Rosenthal’s canal without mispositioned SGNs observed in the central auditory nerve pathway across the cochlea (Fig. 6I).

**Figure 7:**
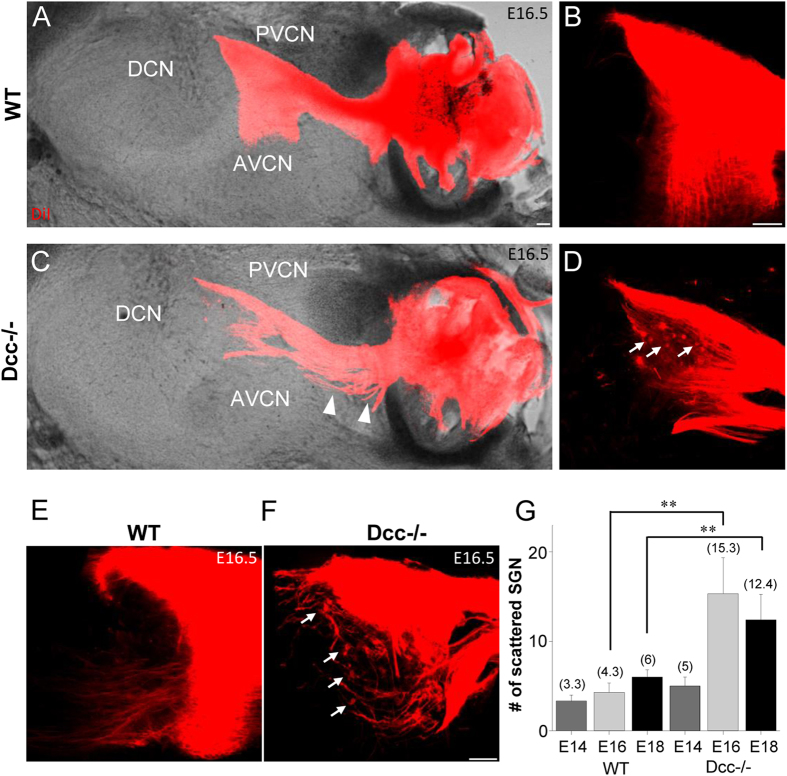
Disorganization of central auditory nerves towards the cochlear nucleus in the Dcc mutant embryo. (**A–D**) DiI-labeled auditory nerve fibers (red) extending from the cochlea to cochlear nucleus in sagittal sections of hindbrains of wild-type and Dcc^−/−^ animals at E16.5, merged with the corresponding DIC image. Images in (**B,D**) are high-magnification views of bifurcated nerve fibers shown in (**A,C**). Arrows point to scattered SGN cell bodies. Arrowhead points to aberrant axon bundles drifting away from the body of central nerve. “DCN”, dorsal cochlear nucleus; “PVCN”, posteroventral cochlear nucleus; “AVCN”, anteroventral cochlear nucleus. (**E,F**) Sagittally sectioned E16.5 cochlear nucleus from another wild-type and Dcc^−/−^ animal, with auditory nerve fibers labeled with DiI. Arrows point to scattered SGN cell bodies. (**G**) Average number of scattered SGN cell bodies in the proximity of auditory axon terminals within the cochlear nucleus from E14.5 to E18.5 for wild-type and Dcc^−/−^ animals. **p < 0.01, t test. N = 5 embryos per group for both genotypes. All scale bars represent 100 μm.

Mispositioning of SGNs was also evident at the terminal region of central auditory nerve axons in the cochlear nucleus (Fig. 7A–D). This abnormality was significant at as early as E16.5 (Fig. 7E–G), similar to the onset of developmental defects in the periphery (Fig. 4I). In addition, there were aberrant fiber bundles deviating from the main trajectory of the central axons, whereas the wild-type central axons exhibited a compact trajectory (Fig. 7A,C). In the wild-type cochlea, the central axons bifurcated, with the ascending and descending branches projecting towards the anterior and posterior parts of the ventral cochlear nucleus respectively^40^ (Fig. 7B). The bifurcation pattern appeared different in the Dcc mutant, with relatively fewer ascending axons into the anterior ventral cochlear nucleus (Fig. 7B,D,E,F). These results demonstrate that the phenotypic defect in the periphery of Dcc mutant cochlea was also duplicated to some extent in the central auditory pathway, with a similar developmental onset. In addition, Dcc may also play a role in regulating bifurcation of the central axons.

### Is Ntn1 the interacting ligand of Dcc in the development of cochlear innervation pattern?

**Figure 8:**
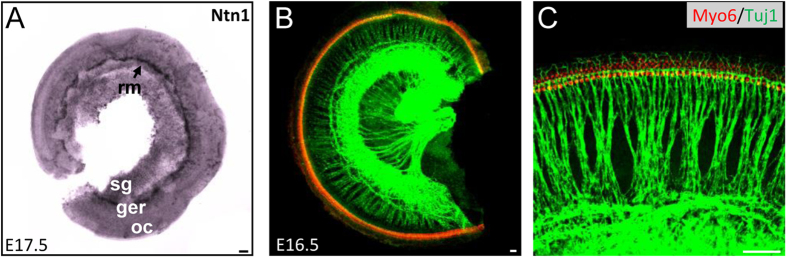
Normal peripheral SG organization in the Ntn1 mutant cochlea. (**A**) *In situ* hybridization of Ntn1 in the whole-mount cochlea at E17.5. “oc”, organ of corti; “ger”, greater epithelial ridge; “sg”, spiral ganglion; “rm”, reissner’s membrane. (**B**) Representative images of whole-mount Ntn1^−/−^ cochlea at E16.5, immunostained with anti-Myo6 (red) and anti-Tuj1 (green) antibodies. (**C**) Higher magnification images of cochleae shown in (**B**). All scale bars represent 50 μm.

Functional interactions between Ntn1 and Dcc to guide CNS axons have been broadly characterized^41^,^42^, and neural defects in Dcc and Ntn1 knockouts usually resemble each other^14^,^43^. We reasoned that if Ntn1 was a ligand for Dcc, it would be expressed by SGNs themselves or in the fiber pathways. By *in situ* hybridization, we found that Ntn1 was not expressed at appreciable levels in the cochlea at E17.5 (Fig. 8A). The strongest expression of Ntn1 transcript was in fact on the Reissner’s membrane (rm) near the SG boundary, part of which remained on the tissue after dissection (Fig. 8A). This expression pattern was consistent with a previous report demonstrating an absence of Ntn1 in the SG, but its presence only in the Reissner’s membrane^44^. Further examination of peripheral auditory circuit development in Ntn1 knockout^43^ cochleae showed no obvious defects of SG innervation patterns or SG assembly as observed in the Dcc mutant (Fig. 8B,C). These results demonstrate that Ntn1 is not present in a spatial manner consistent with a role in regulating SG development via interacting with Dcc, and that different ligands for Dcc are likely responsible.

## Discussion

Our study suggests that Dcc mediates the proper organization of SG assembly and its innervation pattern in the developing auditory circuits. Dcc^−/−^ mice exhibited spatial disorganization of SG cell bodies and fibers in both peripheral and central auditory pathways beginning from E16.5. This distinct developmental time point at which the mispositioning of SGN cell bodies occurs, indicates that the observed phenotypic defects are not due to any abnormality in the initial delamination of neuroblasts from the otocyst when forming the SG aggregate, which occurs at around E10^45^. Although the development of central projections occurs earlier than the peripheral, with the central projections reaching the hindbrain at around E11.5^46^, in the Dcc mutant the central and peripheral auditory projections developed defective phenotypes around the same time. This suggests that Dcc affects a developmental process common for the central and peripheral auditory projections.

Previously we reported mispositioning of SGN cell bodies in Slit2 (and Robo) mutant mice^23^. Although this specific defective phenotype appears similar in Slit2 and Dcc mutants, the developmental contexts that trigger this defect may be different between the two mutants, as the onsets of mis-migration of SGNs are not the same. In the Slit2 mutant, mispositioned SGNs are seen starting from E14.5 when SGNs start to extend their peripheral axons towards the organ of Corti, while in the Dcc mutant mispositioning of SGNs is only observable starting from E16.5. Based on the currently known cellular processes during mouse cochlear development, there exists a possibility that the defect in the Dcc mutant is linked to convergent extension, which also begins at around E16^47^. During the convergent extension process, the cochlear duct extends, resulting in significant changes in cochlear length and width^47^,^48^. SGNs are inevitably engaged in this process as they elongate and migrate with the growing cochlear duct. Rearrangements of the cytoskeleton and cellular interactions via certain surface molecules are required for SGN elongation and migration^49^. The whole process would most likely bring a physical tension upon the assembly of SGNs. Possibly, in the Dcc mutant, the SGN intercellular interactions become less effective to overcome this tension, resulting in the partial disassembly of SG. If this were to be the case, potential Dcc-interacting ligands, possibly also expressed in SGNs, may increase the adhesion between SGNs. This is reminiscent of a previous report that Dcc can mediate the adhesion of spinal commissural neurons to the substrate via substrate-bound netrin-1 proteins^50^. We thus postulate that Dcc is involved in maintaining strong SGN interactions for a proper confinement of their cell bodies within the Rosenthal’s canal.

Although the neurites extended from the mispositioned SGNs contributed to the overall pattern of disorganized SG fibers in the periphery, a significant portion of defective fibers seemed to originate directly from the SG (Fig. 2H,I). This suggests that there is a direct effect of Dcc signaling on axon guidance. In addition, there could be a secondary effect from SGN mispositioning on fiber misrouting, since the mislocation may result in a failure of proper spatial and temporal exposure to axon guidance cues. Further investigations will be needed to elucidate mechanisms behind Dcc signaling in the organization of SGN fibers.

All the ectopic SGNs in the periphery and CNS were mis-migrated along the auditory nerves or were largely confined to the nerve pathway. The misrouted SG fibers, on the other hand, were less spatially restricted compared with SGN cell bodies, as they were scattered throughout the cochlea. This difference in distribution pattern between mislocated cell bodies and fibers may be due to limited routes by which SGN cell bodies can travel through. Previous studies have revealed that the cochlea is consisted of a web of connective tissue fibers interwoven into a porous meshwork, which is suitable for the exchange of fluids and molecular substances^51^,^52^. SGN fibers may have been able to pass through these connective tissues, whereas SGN cell bodies may not.

Since Ntn1 expression site is not even close to SGNs and their fibers, it is unlikely that Ntn1 can play a significant role in the development of SGNs and their fibers. Nonetheless, we cannot exclude the possibility that Ntn1 can interact with Dcc in mediating other aspects of cochlear development, e.g. the morphological development of cochlear ducts. Future work is required to identify the molecular partner of Dcc underlying its regulation of SG organization and their fibers. Based on the extremely weak expression of Ntn1 in the cochlea, it is likely that molecules other than Ntn1 serve as Dcc ligands. In fact, other secreted molecules such as Draxin and CBLN4 have recently been reported to bind to Dcc^53^,^54^. Whether these molecules can serve as Dcc-interacting partners in SG development awaits to be tested in the future.

As previous studies demonstrate that hearing loss can be caused by a reduction in synaptic innervation of hair cells^55^,^56^, the diminished ribbon synapses observed in the Dcc mutant raises the possibility that Dcc mutations are underlying some cases of sensorineural hearing loss, which is of great interest to be further validated. Because Dcc^−/−^ animals are embryonic lethal, we could not perform hearing tests on these animals. Perhaps Dcc^Kanga^ strain^4^ can be utilized to test the direct connection between hearing loss and Dcc, since the homozygous survives into the adulthood and shows similar brain defects as those reported for the targeted allele of Dcc^4^.

Overall, our results suggest that Dcc plays a role in regulating the proper spatial patterning of SGNs during cochlear development and may contribute to sensorineural hearing loss phenotypes. In the future, it will be of particular interest to examine human patients with mirror movements for hearing deficits.

## Methods

### Animals

Mice were handled according to the protocols approved by the Institutional Animal Care and Use Committees at the University of Southern California (USC). The *Dcc* (*Dcc*^tm1Wbg^)^14^ mouse line was maintained on C57BL/6 background. Dcc homozygous mutants were obtained by crossing heterozygous littermates with the plug day designated as E0.5. *Dcc*^+/−^ and *Dcc*^−/−^ embryos were genotyped by genomic PCR (gPCR) using wild-type forward (5′ggccattgaggttccttt3′), wild-type reverse (5′aagacgaccacacgcgag3′) and mutant reverse (5′tcctcgtgctttacggtatc3′) primers. Ntn1 mutant^43^ embryos were obtained from Dr. Le Ma.

### Tissue dissection, FACS, RNA amplification, and Microarray and RNA sequencing

Preparation of RNA sequencing samples was performed as described previously^26^. P3 to P7 mouse cochleae from Gad1-GFP mice for the purification of SCs in the GER region and PV-Cre;Ai6 mice for the purification of HCs and SGNs were dissected out. SCs are positive for GFP in the Gad1-GFP line, and HCs and SGNs are positive for GFP in PV-Cre;Ai6 mice. The GFP-labeled HC and SGN regions were mechanically separated during the cochlear dissection as there is a clear structural separation between the two regions. The isolated tissues were treated with activated papain (20U/ml; Worthington) for 20 min followed by 2 min crude typsin (0.5 mg/ml, Sigma-Aldrich) to achieve complete dissociation. Then, fluorescence-activated cell sorting (FACS) was performed at the Flow Cytometry Core Facility of USC. Cell suspensions were fed into a BDAriaII sorter and purified using 488 nm laser excitation and a 100-μm cytoNozzle. 2000 cells for each distinct cell population were collected into DMEM plus 10% FBS and pelleted down through centrifuge. RNA was extracted from the collected cells using PicoPure RNA isolation kit (Arcturus). Each RNA sample was then amplified using WT-Ovation Pico amplification kit (Nugen) and sequenced with Illumina HiSeq 2000 (Illumina) at the USC Genomics Center following the manufacturer’s instructions. Microarray samples were prepared from P5 to P7 mouse cochleae as described previously^23^. HCs were purified with FASC after staining wildtype cochleae with 5 μM FM1-43 (Invitrogen) or after dissecting tdTomato-labelled HC regions from PV-Cre;Ai14 (with SG trimmed off) mice. There were no differences in fluorescence expression pattern in the cochlea between PV-Cre;Ai14 and PV-Cre;Ai6 animals. For the SGN population, fluorescent SGNs from PV-Cre;Ai14 cochleae, in which the HC region was mechanically trimmed off, were collected. Preparation of RNA samples was similar to that for the RNA-sequencing experiments. Samples were profiled on GeneChip Mouse Genome 430 2.0 Array (Affymetrix) and analyzed within R environment.

### Immunohistochemistry

For whole-mount beta-III Tubulin (Tuj-1), Myosin6 (Myo6), C-terminal binding protein 2 (CtBP2), Cleaved Caspase-3 (CC3), and Parvalbumin (PV) staining, mouse heads at desired time points were fixed with 4% PFA for 2–24 hours. Fixed cochleae were dissected out and permeabilized with 0.5% TrionX-100 followed by incubation in 10% serum blocking buffer for at least 1 hour at room temperature. Incubation of primary antibody was carried out for overnight at 4 °C, followed by Alexa-conjugated secondary antibodies (1:1000, Invitrogen). For cross-section staining, mouse heads were fixed with 4% PFA for overnight. Fixed inner ear bone structure was dissected out and cryoprotected with sucrose. Tissue was then embedded in OCT (Tissue-Tek) and cut into 35-μm sections using a vibrotome (Leica). Sections were later stained using Tuj-1, Myo6, and PV as the whole-mount staining procedure. Antibodies used in this study and their dilution were as followed: Alexa488-conjugated mouse anti-Tuj1 (1:300; Covance), rabbit anti-PV (1:300; Swant Inc.), rabbit anti-Myo6 (1:500, Millipore), mouse anti-CtBP2 (1:250, BD Biosciences), rabbit anti-CC3 (1:300, Cell Signaling Technology).

### Imaging

Confocal z-stack images were obtained using Fluoview1000 (Olympus), projected using National Institutes of Health (NIH) ImageJ and further processed using Inkscape.

### *In situ* hybridization

*In situ* hybridization was performed as previously described 23. Probes were generated using cDNA probes by RT-PCR. Primer pair for Dcc follows: forward 5^′^-ATGGTGACCAAGAACAGAAGGT-3^′^ and reverse 5^′^-AATCACTGCTACAATCACCACG-3^′^. Primer pair for Netrin-1 follows: forward 5^′^-CTTCCTCACCGACCTCAATAAC-3^′^ and reverse 5^′^-TAGAGCTCCATGTTGAATCTGC-3^′^. After subcloning, the identity of the probe was confirmed by DNA sequencing.

### Dye labeling

Embryonic mouse head was fixed in 4% PFA for at least overnight. Cochlea was exposed by removing the inner ear bones and a small crystal of lipophilic carbocyanin dye 1,1^′^-dioctadecyl-3,3,3^′^,3′-tetramethylindocarbocyanine perchlorate (DiI, Molecular Probes^TM^) was placed in the mid turn of the cochlea and incubated at 37 °C in PBS for 4 days. Cochlear nuclei were then sectioned with a sagittal plane cut of the fixed head at 300 μm thickness to include the entire cochlea nucleus in one section. Dissected pieces of cochlear nucleus were then cleared with Clear^T^^57^ and imaged with confocal microscopy. For DiI labeling of efferent fibers, small piece of DiI crystal was inserted into the olivocochlear fiber pathway and incubated at 37 °C in PBS for 3 weeks. Cochlea was dissected out and imaged whole-mount with confocal microscopy.

### Image analysis and statistics

All the confocal image stacks were processed in Fiji (ImageJ) software and 3D reconstructions were performed using Olympus FV10-ASW 3.1 software. The hair cell layers and boundaries of SG were identified based on the immunostaining of Myo6 and Tuj1, respectively, from the image series of the confocal Z-stack. For calculating area occupancy by fibers, whole-mount immunostained z-stacks from the apex to base of HCs were converted to binary images. Pixels of outlined objects in binary images were then counted and subtracted from the pixel number of the total area. Significance was tested using two-tailed Student’s t-test.

Mispositioned SG cell bodies were identified and quantified based on SGN cell body morphology by DiI labeling or PV/Tuj-1 immunostaining. For quantification of number of mispositioned SGNs along the central auditory pathway, a circular boundary with 200 μm radius was set, with the center positioned at the most medial edge of the Rosenthal’s canal, and any SGNs within this range were counted. For counting ribbon synapses on inner hair cells, whole-mount cochleae stained with anti-CtBP2 were used to acquire confocal z-stacks with 0.3 μm intervals. The total number of ribbon synapses was divided by the total number of IHC nuclei (stained with anti-CtBP2) to obtain the average number of synapses per IHC. Significance was tested using two tailed Student’s t-test.

## Additional Information

**How to cite this article**: Kim, Y. J. *et al*. Dcc Mediates Functional Assembly of Peripheral Auditory Circuits. *Sci. Rep*. **6**, 23799; doi: 10.1038/srep23799 (2016).

## Figures and Tables

**Table 1:** RNA-seq gene expression profile of classical axon guidance molecules in developing mouse cochlea (P3-P7).

Guidance Molecules				Receptors			
Gene Name	Cell Types			Gene Name	Cell Types		
	SC	HC	SGN		SC	HC	SGN
Ntn1	0.291987	0.287536	0.313077	Dcc	0	0	1.27416
Ntn3	0.003005	0	0.00449	Neo1	15.752	5.78751	21.8909
Ntn4	0	0	0	Unc5a	0.171611	0.945418	0
Ntn5	0	0	0	Unc5b	1.69626	3.07132	1.51094
Ntng1	4.92166	1.34048	18.3732	Unc5c	5.83176	0.11426	4.60669
Ntng2	0	0	0	Unc5cl	0	1.10823	0.512023
Slit1	0	0	2.13429	Unc5d	1.01709	0.108628	4.90621
Slit2	23.5068	0.29686	6.81978	Robo1	16.9609	4.86413	5.39499
Slit3	1.23503	0.153293	1.33909	Robo2	23.5354	11.6351	2.0519
Efna1	19.7586	4.56922	11.2321	Robo3	0	0	0
Efna2	1.40287	0.88688	3.973	Robo4	0	0	5.48805
Efna3	0.956779	0	0	Epha1	5.30795	3.16009	0.649002
Efna4	10.7291	1.86627	9.02716	Epha2	0.083158	0	0.846352
Efna5	2.53626	34.7123	22.7668	Epha3	0	0.75091	0.46239
Efnb1	1.27314	0	4.87075	Epha4	18.4469	37.0778	26.3534
Efnb2	36.7231	18.0239	33.9682	Epha5	0.074131	0.343676	2.17597
Efnb3	5.76249	1.82149	13.8246	Epha6	0	0	0.06812
Sema3a	16.6265	6.10089	7.14609	Epha7	172.875	5.87868	12.488
Sema3b	6.72012	0.826158	17.4055	Epha8	0	0	0.188284
Sema3c	14.3912	1.07593	10.8667	Epha10	0	0	0
Sema3d	15.7132	0.238447	10.1495	Ephb1	6.91325	3.8911	3.00102
Sema3e	0.313725	2.3356	27.3714	Ephb2	2.53103	0.731591	3.45582
Sema3f	0.672963	0.091166	0.238233	Ephb3	2.18359	2.27847	2.46585
Sema3g	0	0	0	Ephb4	1.2616	0.318164	1.78494
Sema4a	0.391461	0	0.94313	Ephb6	1.78292	0.656599	2.25204
Sema4b	2.38712	0.413705	0.473522	Plxna1	2.8087	3.36966	2.64267
Sema4c	1.23898	1.81503	1.17214	Plxna2	2.63166	4.87033	2.03184
Sema4d	1.38416	1.18624	0.70749	Plxna3	2.84983	0.868645	3.7259
Sema4f	7.9325	1.13515	1.87143	Plxna4	1.88387	0.651583	0.870651
Sema4g	1.95086	0.635703	3.51307	Plxnb1	6.63703	3.58485	4.18291
Sema5a	1.20719	0.254363	15.7109	Plxnb2	11.8443	4.05841	9.5753
Sema5b	2.23085	31.0786	3.46451	Plxnb3	0.363518	0.390343	3.64299
Sema6a	9.57572	3.83104	6.93643	Plxnc1	4.94658	0.27669	1.49693
Sema6b	0.302393	1.10583	0.244681	Plxnd1	0	0	0.609175
Sema6c	0	0.153224	0	Nrp1	8.11035	0.551931	45.2042
Sema6d	11.0265	1.08001	15.8813	Nrp2	3.8243	15.2802	18.4682
Sema7a	0.139595	0.501136	2.21496				

SC, supporting cell; HC, hair cell; SGN, spiral ganglion neuron.

Numerical values are in Fragments Per Kilobase of exon per Million (FPKM).
